# Public Awareness of Common Age-Related Eye Diseases in Northern Saudi Arabia

**DOI:** 10.7759/cureus.56841

**Published:** 2024-03-24

**Authors:** Mujeeb Ur Rehman Parrey, Maha M Abdul-Latif, Shmoukh Mushref M Alruwaili, Khulud Hamed Saud Alshammari, Razan Ibrahim Ali Alsayer, Nouf Khalid J Alanazi, Mohamed M Abd El Mawgod

**Affiliations:** 1 Ophthalmology, Surgery, Northern Border University, Arar, SAU; 2 Ophthalmology, Faculty of Medicine, Northern Border University, Arar, SAU; 3 Medicine, Northern Border University, Arar, SAU; 4 Public Health and Community Medicine, Faculty of Medicine, Al-Azhar University, Asyut, EGY

**Keywords:** visual impairment, awareness, saudi arabia, glaucoma, diabetic retinopathy, cataract, age-related macular degeneration

## Abstract

Background*: *Visual impairment and blindness pose substantial public health challenges in Saudi Arabia, especially among the elderly susceptible to blinding eye diseases. Assessing awareness of age-related eye diseases (AREDs) is vital for addressing vision loss in this demographic. However, there is a lack of research on ARED awareness in Northern Saudi Arabia, underscoring the need for evidence-based data from adult populations to craft effective health promotion strategies.

Methods: This population-based descriptive survey was conducted on 411 Saudi adults aged 18-70 residing in Arar City, Saudi Arabia, over six months from September 2023. Random sampling was employed, and awareness levels were assessed using a pre-designed questionnaire. Data analysis was performed using Statistical Product and Service Solutions (SPSS, version 20.0; IBM SPSS Statistics for Windows, Armonk, NY).

Results: Among the 411 participants, 225 (54.7%) were aged 18-29 years, 312 (76%) were females, and 299 (72.6%) held a bachelor's degree or diploma. Regarding awareness, 71.3% knew about cataracts, but nearly half erroneously believed it could be treated solely medically. For diabetic retinopathy (DR), 366 (89%) recognized lifestyle modifications, and 378 (92%) understood screening as preventive measures. Most participants understood prevention methods for all four diseases, but misconceptions about treatment options were observed. Awareness of glaucoma and age-related macular degeneration (ARMD) was lower. No significant age-related differences were found in awareness, except for cataracts (P = 0.001). Education significantly influenced awareness of cataracts, DR, and glaucoma (P = 0.001, 0.013, and 0.008, respectively), but not ARMD (P = 0.606). The study found that the internet is the primary source of information on AREDs for most participants, except for cataracts, where friends and relatives are prominent.

Conclusion: The study reveals varying awareness levels of AREDs among Saudi adults. Although most participants understood preventive measures, misconceptions about treatment underscore the need for accurate education channels. Healthcare professionals must ensure information reliability to effectively combat misinformation and enhance awareness of AREDs.

## Introduction

Vision loss among the elderly is a significant healthcare issue, with approximately one in three elderly individuals experiencing some form of vision-reducing eye disease by the age of 65 [1]. The elderly population in developed countries is rapidly increasing. The four primary causes of vision impairment in the elderly are cataracts, diabetic retinopathy (DR), glaucoma, and age-related macular degeneration (ARMD) [2-4]. ARMD involves the loss of central vision, while primary open-angle glaucoma leads to optic nerve damage and visual field loss. Regular screening examinations are recommended for elderly patients, as glaucoma may initially be asymptomatic. Although cataracts commonly impair vision among the elderly, surgery often proves effective in restoring vision. DR may manifest in the elderly at the time of diagnosis or within the initial years of diabetes onset, necessitating eye examinations with dilation upon diabetes diagnosis and annually thereafter.

Preventive and curative eye care interventions can avert around 80% of blindness and 85% of moderate to severe visual impairment [5]. Blinding eye diseases are more prevalent among elderly populations, and many acquire detailed knowledge about eye conditions while seeking eye care [6]. The lack of awareness regarding these eye conditions poses a significant obstacle to public health strategies and is linked to poorer outcomes in prevention, eye care utilization, and treatment [7-10].

Various studies assessing knowledge of eye care and diseases have been conducted globally and have laid the groundwork for eye health promotion initiatives [11-13]. Misconceptions regarding specific blinding eye diseases have been explored in Arab countries, including Saudi Arabia [14,15]. However, public awareness regarding common eye diseases among Saudi adults has only been studied in Riyadh City [16]. Awareness of common AREDs and their treatment can encourage timely eye care seeking and reduce the burden of visual loss due to AREDs.

Therefore, assessing public awareness and knowledge of AREDs is crucial for developing effective eye care strategies to improve overall eye health. Increased awareness among the population can prompt early medical advice from ophthalmologists, leading to better outcomes and vision loss prevention from common AREDs. Evaluating awareness levels can provide baseline information useful for guiding health promotion initiatives in Saudi Arabia.

The current study aims to assess awareness levels regarding AREDs among Saudi adults and explore existing misconceptions about eye health in the community.

## Materials and methods

Study design: This is a population-based, descriptive cross-sectional study.

Ethical consideration: Ethical approval no. 49/44/H was obtained from the Local Committee of Bioethics at NBU on 05/07/2023.

Research tool: The level of awareness was assessed using a predesigned questionnaire validated by the Ophthalmology Department at Northern Border University, Arar, Saudi Arabia. The questionnaire comprised of three parts: demographic details collection, 10 yes/no awareness questions, and identification of information sources. Participants were allowed to select multiple options for information sources. Free informed consent was obtained from the participants after discussing the idea and objectives of the study. The study was carried out in Arar City from September 2023 to February 2024.

Sampling method and study sample: The convenient sampling method was used. The minimum required sample size of 386 adults was determined utilizing Epi info (version 7.1.5; Centers for Disease Control and Prevention, Atlanta, GA). This calculation was predicated on an anticipated awareness level of 50%, a precision of 5%, and a confidence level of 95%.

Inclusion and exclusion criteria: Both genders who are 18 years and above were included. Individuals who lived outside the study area and who were mentally challenged were excluded.

Data analysis: The collected data were coded and analyzed on Statistical Product and Service Solutions (SPSS, version 20; IBM SPSS Statistics for Windows, Armonk, NY). The effect of the different demographic characteristics on the level of awareness was analyzed using the chi-square test. Significance was considered with a P-value less than 0.05.

## Results

A total of 411 participants returned fully completed questionnaires. Among them, 225 (54.7%) fell into the 18-29 age group, while 102 (24.8%) were aged between 40 and 49. Female participants made up 312 (76%) of the sample, and 299 (72.6%) had achieved a bachelor's degree or diploma. A detailed breakdown of demographic data is provided in Table 1.

**Table 1:** Participants’ demographic data

Items	Number	Percent
Age		
18-29	225	54.97
30-39	43	10.4
40-49	102	24.8
50-59	33	8.0
60-69	8	1.9
Gender		
Male	99	24.0
Female	312	76.0
Number of family members		
1	6	1.5
2	14	3.4
More than 2	392	95.1
Education Level		
Illiterate	1	.2
Primary	1	.2
Intermediate	13	3.2
High school	60	14.6
Bachelor/diploma	299	72.6
Master/Ph.D.	38	9.2
Job Status		
Student	192	46.6
Employee	162	39.3
Nin-employee	42	10.2
Retired	16	3.9
History of Chronic Illnesses		
Hypertension	20	4.9
Diabetes	24	5.8
Thyroid disease	23	5.6
Rheumatoid arthritis	28	6.8
Others	317	76.9
Personal History of Eye Disease		
Yes	115	27.9
No	297	72.1
Family History of Eye Disease		
Yes	145	35.2
No	267	64.8

Participants exhibited varied responses to awareness questions regarding the four common age-related diseases. While 293 (71.3%) of participants were aware of cataracts, nearly half incorrectly believed that cataracts could be treated solely with medical intervention. Concerning DR, the majority correctly identified lifestyle modifications and screening as preventive measures (366, 89%, and 378, 92%, respectively). It is encouraging to note that a significant majority of the participants, over 86% each, have a solid understanding of prevention methods for all four diseases. However, it is concerning to observe that there were misconceptions regarding treatment options for all these conditions. The level of awareness about glaucoma and ARMD was lower, with only 237 (57.7%) and 254 (61.8%) of participants, respectively, being aware of these conditions. Individual responses to awareness questions are detailed in Table 2.

**Table 2:** Awareness of age-related eye diseases (AREDs) in participants (n=411) The data are presented as n, %. ARMD: Age-related macular degeneration

Survey Question Response	Cataract		Diabetic Retinopathy		Glaucoma		ARMD	
	Yes, n (%)	No, n (%)	Yes, n (%)	No, n (%)	Yes, n (%)	No, n (%)	Yes, n (%)	No, n (%)
Have you heard about this disease?	293 (71.3)	118 (28.7)	256 (62.3)	155 (37.7)	237 (57.7)	174 (42.3)	254 (61.8)	157 (38.2)
Can this disease lead to blindness?	264 (64.2)	147 (35.8)	297 (72.3)	114 (27.7)	304 (74.0)	107 (26.0)	361 (87.8)	50 (12.2)
Are regular checkups important for this disease?	359 (87.3)	52 (12.7)	36 (89.3)	44 (10.7)	362 (88.1)	49 (11.9)	195 (47.4)	216 (52.6)
Is medical treatment alone sufficient for this disease?	202 (49.1)	209 (50.9)	177 (43.1)	234 (56.9)	185 (45.0)	226 (55.0)	244 (59.4)	167 (40.6)
Is surgical treatment alone necessary for this disease?	230 (56.0)	181 (44.0)	165 (40.1)	246 (59.9)	185 (45.0)	226 (55.0)	346 (84.2)	65 (15.8)
Are both medical and surgical treatments required for this disease?	341 (83.0)	70 (17.0)	310 (75.4)	101 (24.6)	316 (76.9)	95 (23.1)	182 (44.3)	229 (55.7)
Is lifelong medication necessary for this disease?	197 (47.9)	214 (52.1)	280 (68.1)	131 (31.9)	258 (62.8)	153 (37.2)	387 (94.2)	24 (5.8)
Is public awareness important for the prevention of this disease?	382 (92.9)	29 (7.1)	387 (94.2)	24 (5.8)	375 (91.2)	36 (8.8)	356 (86.6)	55 (13.4)
Can change in lifestyle help in the prevention of this disease?	348 (84.7)	63 (15.3)	366 (89.1)	45 (10.9)	336 (81.8)	75 (18.2)	379 (92.2)	32 (7.8)
Can screening help in the prevention of this disease?	375 (91.2)	36 (8.8)	378 (92.0)	33 (8.0)	365 (88.8)	46 (11.2)	365 (88.8)	46 (11.2)

No significant differences in awareness were found between various age groups concerning cataracts, DR, and glaucoma. However, there was a significant difference in awareness related to cataracts across different age groups (P-value = 0.001). Similarly, the level of education significantly influenced awareness regarding cataracts, DR, and glaucoma (P-values = 0.001, 0.013, and 0.008, respectively), but not ARMD (P-values = 0.606). Further associations are presented in Table 3.

**Table 3:** Age-related eye diseases' (AREDs) awareness and association with participants’ demographic variables The data have been represented as n, %, and p-value < 0.05 is considered significant. ARMD: Age-related macular degeneration *Significant

Variable		Cataract			Diabetic Retinopathy			Glaucoma			ARMD		
		Yes, n (%)	No, n (%)	P	Yes, n (%)	No, n (%)	P	Yes, n (%)	No, n (%)	P	Yes, n (%)	No, n (%)	P
Age	18-29	144 (49.1)	81 (68.6)	0.001^*^	132 (51.6)	93 (60.0)	0.239	132 (55.7)	93 (53.4)	0.057	134 (52.8)	91 (58.0)	0.827
	30-39	29 (9.9)	14 (11.9)		25 (9.8)	18 (11.6)		19 (8.0)	24 (13.8)		26 (10.2)	17 (10.8)	
	40-49	83 (28.3)	19 (16.1)		68 (26.6)	34 (21.9)		59 (24.9)	43 (24.7)		67 (26.4)	35 (22.3)	
	50-59	30 (10.2)	3 (2.5)		25 (9.8)	8 (5.2)		19 (8)	14 (8)		22 (8.7)	11 (7.0)	
	60-69	7 (2.4)	1 (0.8)		6 (2.3)	2 (1.3)		8 (3.4)	0 (0)		5 (2.0)	3 (1.9)	
Gender	Male	70 (23.9)	29 (24.6)	0.883	62 (24.2)	37 (23.9)	0.936	58 (24.5)	41 (23.6)	0.831	63 24.8)	36 (22.9)	0.666
	Female	223 (76.1)	89 (75.4)		194 (75.8)	118 (76.1)		179 (75.5)	133 76.4)		191 (75.2)	121 (77.1)	
Education Level	Illiterate	1 (0.3)	0 (00.0)	0.001^*^	1 (0.4)	0 (00.0)	0.013^*^	1 (0.4)	0 (00.0)	0.008^*^	1 (0.4)	0 (00.0)	0.606
	Primary	2 (0.7)	11 (9.3)		2 (0.8)	11 (7.1)		1 (0.4)	12 (6.9)		6 (2.4)	7 4.5	
	Intermediate	36 (12.3)	24 (20.3)		36 (14.1)	24 (15.5)		33 (13.9)	27 (15.5)		34 (13.4)	26 16.6	
	High school	220 (75.1)	78 (66.1)		190 (74.2)	108 (69.7)		178 (75.1)	120 (69.0)		189 (74.4)	109 (69.4)	
	Bachelor/diploma	33 (11.3)	5 (4.2)		26 (10.2)	12 (7.7)		23 (9.7)	15 (8.6)		23 (9.1)	15 (9.6)	
	Master/Ph.D.	1 (0.3)	0 (00.0)		1 (0.4)	0 (00.0)		1 (0.4)	0 (00.0)		1 (0.4)	0 (00.0)	

**Figure 1:**
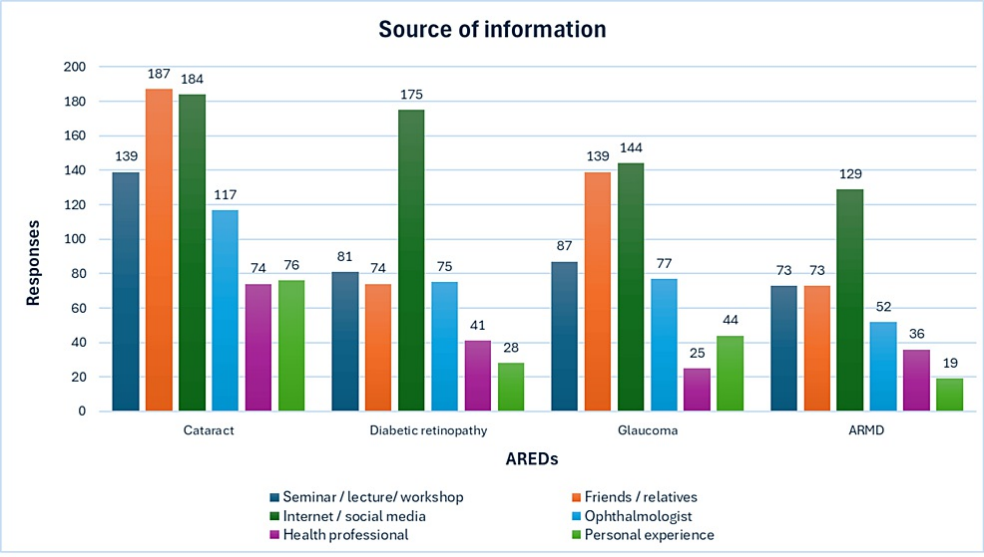
Participants’ source of information about age-related eye diseases (AREDs) The data are presented as participants' number of responses for each ARED in relation to the source of information.

The primary sources of information regarding AREDs among participants were found to be the internet, except in the case of cataracts, where friends and relatives were the predominant sources. For cataracts, the immediate secondary source was the internet, while for glaucoma and ARMD, it was friends and relatives. Conversely, information about DR mainly came from ophthalmologists. The complete data about the source of information are depicted in Figure 1.

## Discussion

The study revealed both positive and concerning aspects of participants' awareness regarding AREDs. Despite a considerable percentage of participants being aware of cataracts, a significant proportion incorrectly believed that cataracts could be treated solely with medical intervention. The awareness about glaucoma and ARMD was comparatively lower. The poor levels of awareness about glaucoma and ARMD are in line with the findings of studies conducted in other countries [17-19].

While a significant majority demonstrated a basic understanding of disease prevention strategies, misconceptions regarding treatment options were prevalent across all conditions. This is consistent with a study that reports similar misconceptions about AREDs [20]. This suggests a need for targeted educational interventions to address these gaps in knowledge.

The study found significant associations between demographic factors such as age and education level with awareness levels. While no significant differences were observed in awareness across different age groups for most conditions, education level significantly influenced awareness regarding cataracts, DR, and glaucoma. This highlights the importance of tailoring educational campaigns to different demographic groups to effectively address knowledge gaps. Younger participants may require targeted educational efforts to improve awareness of certain eye conditions. Numerous studies have confirmed gaps in awareness regarding AREDs [17,20-22].

The primary sources of information regarding AREDs among participants were found to be the internet, except in the case of cataracts, where friends and relatives were the predominant sources. For cataracts, the immediate secondary source was the internet, while for glaucoma and ARMD, it was friends and relatives. Conversely, information about DR mainly came from the internet, followed by ophthalmologists. The study suggests that information obtained from the internet, friends, and relatives may have contributed to the misinformation observed. This highlights the critical importance of effectively utilizing appropriate channels for public health education and awareness campaigns. Moreover, it underscores the necessity of ensuring the accuracy and reliability of information disseminated through these sources to counteract misinformation effectively. Thus, healthcare professionals should play a crucial role in providing accurate information and guiding individuals toward trustworthy resources for informed decision-making regarding their eye health.

The findings of the study have important implications for public health interventions aimed at promoting awareness and understanding of age-related diseases. Effective educational campaigns should focus not only on preventive measures but also on dispelling misconceptions about treatment options. Targeting specific demographic groups based on factors such as age and education level can help maximize the impact of these interventions.

Limitations

The reliance on self-reported data and potential biases inherent in survey-based research should be acknowledged as one of the primary limitations of this study. Furthermore, future studies could explore additional factors influencing awareness levels, such as socioeconomic status and access to healthcare.

## Conclusions

The current study reveals varying awareness levels of AREDs among surveyed Saudi adults. While cataracts garnered relatively high awareness, knowledge about glaucoma and ARMD was lower. Misconceptions persisted, notably regarding cataract treatment options. Education significantly influenced awareness, particularly for cataracts, DR, and glaucoma, with higher education correlating with greater awareness. While age did not significantly affect awareness for most diseases, cataract awareness varied across age groups. Targeted educational efforts may be necessary for younger participants. However, participants demonstrated a basic understanding of the preventive measures for all four AREDs. The internet was a primary information source. Addressing knowledge gaps and misconceptions through tailored interventions could enhance eye health outcomes in the population.

## Figures and Tables

**Figure 2:**
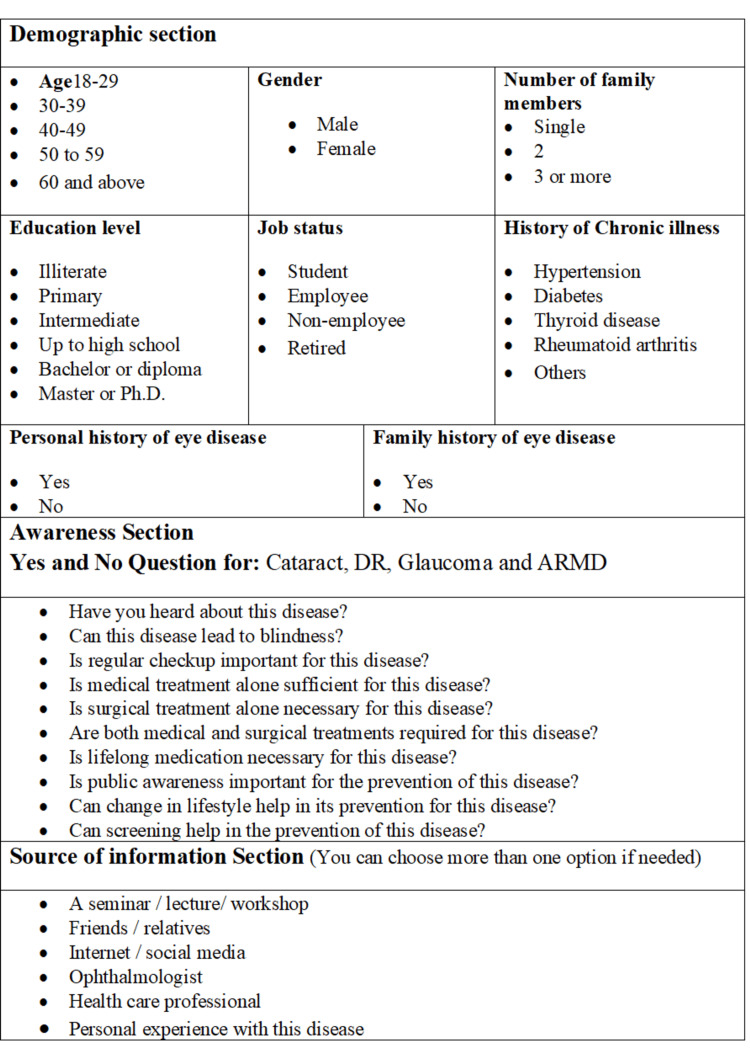
Questionnaire on public awareness of common age-related eye diseases in Northern Saudi Arabia
